# Editorial: Innovations in recovery science: pathways, policies, and platforms that promote thriving after addiction

**DOI:** 10.3389/fpubh.2025.1731271

**Published:** 2025-11-24

**Authors:** Ashli J. Sheidow, Bettina B. Hoeppner, Robert P. Pack, George Jay Unick, Kenneth Blum

**Affiliations:** 1 Lighthouse Institute, Chestnut Health Systems, Bloomington, IL, United States; 2 Department of Psychiatry, Massachusetts General Hospital, Boston, MA, United States; 3 Harvard Medical School, Boston, MA, United States; 4 East Tennessee State University, Johnson City, TN, United States; 5 University of Maryland School of Social Work, Baltimore, MD, United States; 6 Western University of Health Sciences, Pomona, CA, United States

**Keywords:** recovery science, recovery community centers, peer recovery support services, recovery housing, substance use disorders

Nearly 50 million people in the U.S. have a current substance use disorder (SUD) (1, 2), with hundreds of fatalities daily due to drug and alcohol use (3, 4). The systems and services for addressing substance use conditions are fragmented, difficult to access, and insufficiently understood. Contrary to popular belief, however, over 70% of individuals with a history of substance use issues report recovering successfully (1, 2). Recovery is not only possible, it is likely.

Much remains to be learned about recovery processes and recovery support services. The individualized nature of recovery journeys has only nascent literature, limiting effective service development. For recovery support services, there is little research about their operation, network structures, payment methods, measurement criteria, workforce preparation, and more; in fact, a recent review of the recovery-supportive interventions literature from 2000 to 2023 showed only 25 studies (5). Recovery science redirects focus from only treating SUDs toward supporting thriving, long-term recovery across social, psychological, and structural domains. The *Innovations in Recovery Science: Pathways, Policies, and Platforms that Promote Thriving After Addiction* Research Topic responds to this imperative by collecting empirical, theoretical, methodological, and policy-oriented contributions on how individuals move through recovery trajectories, how systems can better support those trajectories, and what platforms amplify recovery success. It highlights new research, practices, and innovations that inform sustainable recovery outcomes and future interventions.

With 24 articles, this is the largest ever Research Topic on recovery science, showing the field's burgeoning research activity. It includes 17 original research papers, three high-quality reviews, three conceptual analyses, and one case study that examine recovery as a dynamic process and present novel conceptual models, interventions, measurement strategies, system-level reforms, and community-centered approaches. Using the recovery support services categories of Day et al. (6), seven papers specifically focus on Recovery Community Centers (RCCs), six on Peer Recovery Support Services (PRSS), four on recovery housing, and one on continuing care; not represented are employment services and educational settings. Beyond recovery services, nine focus on recovery processes (some papers are in multiple categories). The 24 contributions cluster into themes representing key categories and topics in recovery science: 1. RCCs; 2. PRSS and Workforce; 3. Recovery Housing; 4. Recovery Processes and Identity; 5. Family and Special Populations; 6. Policy and Systems Innovation; and 7. Measurement and Assessment Innovations.

## Recovery Community Centers (RCCs)

RCCs play a central role in providing accessible, community-based recovery supports. Hoeppner, Williamson, Nicoll, et al. examined the value of and barriers to linkages between opioid treatment programs (OTPs) and RCCs. RCCs' reach into diverse populations has been highlighted as a key asset (7); DeCristofaro et al. expanded on this, examining if RCC implementation differed depending on the racial/ethnic composition of communities served, finding the RCC model to be robust. The daily experiences of RCC participants further illustrate the nuanced ways these centers shape recovery; Apsley et al. captured participant-reported helpfulness, meaningfulness, and recovery identity trajectories via a daily diary design, emphasizing how engagement at RCCs translates into personal recovery processes. Improving valid measurement for the impact of RCCs-necessary for effectiveness trials–Hoeppner, Williamson, Simpson, et al. sought input from RCC directors nationwide to build a consensus on outcome measures for future trials. Additional papers included a focus on the RCC setting, but are discussed within other themes (Castedo de Martell et al.; Feld et al.; Lancaster et al.).

## Peer Recovery Support Services (PRSS) and workforce

PRSS are critical in both formal and informal recovery networks. Drazdowski et al. examined the challenges and advances in supporting linkage facilitators who help individuals navigate recovery systems. Bell et al. explored workforce outcomes among peer supports, revealing how individual and organizational factors shape peer retention and effectiveness. Hagaman et al. assessed prevalence of PRSS activities across nine states, highlighting variability in peer integration across settings. The economic dimension of peer-driven interventions was addressed by Castedo de Martell et al., who developed a cost-effectiveness calculator, providing tools for scaling peer programs efficiently. Horn et al. contributed a taxonomy to establish a common language for characterizing PRSS, further supporting workforce development and operational clarity. These workforce-focused studies underscore the infrastructure needed to sustain high-quality PRSS. Lancaster et al. included recovery support meetings as a moderator, but is described in the Recovery Processes and Identity theme.

## Recovery housing

Recovery housing provides safe, structured environments that support residents in sustained recovery. Mericle et al. offered a comprehensive roadmap for maximizing recovery housing effectiveness, including considerations for staff support, community integration, and evidence-based practices. Hibbard et al. combined community-based participatory research (CBPR) and scoping review methods to identify on-the-ground research priorities, ensuring that housing strategies are informed by lived experience. Zoschke et al. demonstrated strategies for sustaining housing operations through diverse funding sources, partnerships, and rent models. Dewey et al. highlighted the role of “wing leaders”, staff who provide individualized support to criminal legal system-involved residents prescribed medications for opioid use disorder (MOUD), further illustrating the importance of tailored staffing in recovery housing.

## Recovery processes and identity

Psychological processes and identity play a pivotal role in sustaining recovery. Lancaster et al. employed within-person designs to explore how social contexts influence recovery identity. Lewis et al. examined the dualistic model of passion in addiction recovery, highlighting motivational dynamics. Heinrich et al. studied psychological safety in The Phoenix, a sober-active community, demonstrating its mediating role in attendance and recovery outcomes. Apsley et al. provided complementary evidence on recovery identity development through daily diary assessments, highlighting how engagement in RCCs fosters meaningful recovery-related self-concepts. Szlyk et al. conducted a qualitative exploration of stigma and interpersonal/intrapersonal influences, showing how societal and self-perceptions can affect recovery. Lewandrowski et al. focused on complex functions of brain reward circuitry and how modulating dopamine could be a part of the recovery process, including the possibility of a short-term blockade followed by long-term dopaminergic upregulation. Cioffi et al., Porter et al., and Vose-O'Neal et al. also examine recovery processes, but are described in the Special Populations theme.

## Family and special populations

Recovery experiences are majorly shaped by life stage, personal characteristics, and family involvement. Examining processes for special populations can be elucidating. Cioffi et al. examined the needs of postpartum emerging adults with SUDs, emphasizing tailored interventions for this population. Porter et al. provided insights from concerned significant others, showing how family engagement influences young adult recovery trajectories. Feld et al. explored reproductive and perinatal health integration with recovery supports, illustrating service adaptation for maternal health. Vose-O'Neal et al. documented recovery pathways in a Black community, highlighting culturally relevant processes. DeCristofaro et al. (also described in the RCC theme) is also relevant here, highlighting how RCCs serving diverse communities contribute to equitable recovery supports.

## Policy and systems innovation

Macro-level levers, policy frameworks, and biomedical innovations are critical for system-wide recovery support. Gaumond et al. presented perspectives from a federal interagency workgroup, outlining research priorities and opportunities for advancing recovery science. Castedo de Martell et al. offered an economic evaluation tool for peer-driven interventions, bridging micro-level services with macro-scale cost-effectiveness considerations. Hoeppner, Williamson, Nicoll, et al. highlighted linkages between OTPs and RCCs, illustrating how integration can enhance recovery ecosystems. Watson et al. explored Recovery Management Checkups implemented in primary care settings at a Federally Qualified Health Center, demonstrating strategies for early intervention and sustained recovery support. Together, these works illustrate how macro-level policies, economic tools, and cross-system innovations intersect with service delivery to enhance recovery outcomes at scale.

## Measurement and assessment innovations

Measurement and assessment advancements are important for recovery science to proceed, offering methods to quantify and understand recovery processes. While all of these are described above, they warrant mention under this theme. Hoeppner, Williamson, Simpson, et al. and Horn et al. developed metrics for RCC effectiveness and a PRSS taxonomy, respectively. Hibbard et al. combined CBPR and scoping review methods to identify and study recovery housing research priorities. Castedo de Martell et al. developed a cost-effectiveness calculator for peer-driven interventions, supporting economic decision-making, but also did this using CBPR which is novel within economic research. These articles provide methodological advances that refine how recovery outcomes, service effectiveness, and economic considerations are assessed, enabling more actionable results.

## Cross-cutting themes

There are several noteworthy cross-cutting themes that reveal key insights.

- **Equity and population tailoring:** Several studies highlight the importance of ensuring recovery supports reach diverse populations. Life-stage considerations are emphasized as critical for tailoring interventions. Stigma-informed approaches further ensure that recovery services are accessible and culturally responsive to those most marginalized.
- **Workforce and peer infrastructure:** The research underscores the pivotal role of PRSS workers and linkage facilitators. Taxonomies of peer roles, prevalence studies, and workforce outcome assessments help standardize practice and improve training. Staffing models in recovery housing illustrate how structured peer involvement can enhance engagement and recovery outcomes.
- **Measurement and methods innovation:** Innovative measurement approaches, including taxonomies, within-person or daily diary designs, and RCC-specific metrics, allow for a more precise understanding of recovery trajectories. Economic evaluation tools complement these methods, providing actionable data on cost-effectiveness and scalability. Together, they advance both the rigor and practical relevance of recovery research.
- **Integration and systems:** Several contributions explore how recovery supports are embedded within broader systems of care. OTP to RCC linkages, primary care Recovery Management Checkups, and federal coordination illustrate opportunities for integration across levels. Evaluations of system-level strategies also inform cost-effective approaches for scaling services nationally.
- **Sustainability and financing:** Recovery housing and RCC programs require robust funding structures to maintain long-term viability. Studies highlight diverse funding streams, strategic partnerships, and economic evaluation strategies as mechanisms to support sustainability. Understanding these elements is essential for programs to continue serving communities effectively over time.
- **Recovery as social and identity processes:** Recovery is not only a clinical outcome but also a social and identity-driven process. Research on psychological safety, dualistic models of passion, and recovery identity trajectories illuminates how personal and relational factors influence sustained recovery. Family and significant-other engagement further reinforces these processes.

## Challenges and future directions

Despite the substantial contributions of the studies in this Research Topic, several critical challenges remain. The complexity of recovery trajectories, particularly across diverse populations, underscores the need for research attentive to social context and individual experiences. Recovery unfolds nonlinearly over time in response to life events, highlighting the need for flexible modeling approaches to capture these dynamics. Emerging adults represent a key population in this regard, as they exhibit the highest SUD rates (i.e., 7.8% of 12–17 year olds, 25.9% of 18–25 year olds, and 16.4% of adults over 25) (8), yet remain underrepresented in recovery research. Notably, the Research Topic provides some of the first rigorous insights into how recovery supports can be tailored for emerging adults (e.g., Cioffi et al.), highlighting opportunities and gaps. Individuals living in rural communities also experience unique barriers to accessing recovery support services, representing a population that warrants attention.

A persistent challenge is ensuring recovery research is conducted with and led by individuals with lived experience. Incorporating experiential expertise into study design, implementation, and interpretation enables the field to identify meaningful solutions more quickly. Lived experience informs what outcomes matter most, how services can be improved, and how structural and social barriers can be addressed in ways purely academic perspectives might overlook.

Looking forward, the field must expand populations studied and scope of recovery supports examined. A critical need exists for research integrating peer recovery roles, housing, community-based programs, and measurement innovations, while also emphasizing equity, accessibility, and sustainability. Econometric and policy modeling approaches could be leveraged to forecast both desired and unintended consequences of interventions, supporting more informed decision-making.

Translational pathways to practice are essential for maximizing the impact of research. Researchers, practitioners, peers, and community organizations must align to ensure that evidence-based recovery supports reach the intended populations. This requires not only dissemination of findings but also the development of practical toolkits, structured training programs, adaptable knowledge-sharing platforms, and incentive structures for uptake. Effective translation also entails ongoing feedback loops, where real-world implementation informs iterative improvements in interventions, training, and policies, ultimately enhancing reach and effectiveness across diverse contexts.

## Conclusions

This Research Topic marks an important inflection point in recovery science. The 24 contributions collectively suggest that thriving after addiction is not merely about symptom remission, but about supporting holistic, sustained recovery across social, psychological, and structural domains. The authorships also reflect the field's multi-disciplinary and community focus, spanning researchers, clinicians, policymakers, and, most importantly, people who deliver and have received recovery support. We believe this is critical for recovery science and have made efforts to create a welcoming field for people with lived experience and varied perspectives (9, 10). By combining rigorous methodology with the insights of individuals who have navigated recovery firsthand, and by attending carefully to translational and policy pathways, future research can accelerate the development of practical interventions that enhance recovery trajectories across the lifespan, particularly for high-risk, underserved, and rural populations.

Centering equity, lived experience, and translational pathways ensures that recovery innovations reach practitioners, peers, and communities, supported by toolkits, training, dissemination platforms, and appropriate incentive structures. Econometric and policy modeling may further help forecast both intended and unintended consequences, guiding scalable, sustainable approaches to recovery support.

We hope this Research Topic energizes ongoing inquiry, collaboration, and impact. The Consortium on Addiction Recovery Science (CoARS), funded by the National Institute on Drug Abuse, exemplifies these efforts by advancing research networking, training and mentoring students and early-career scientists, and building community partnerships (https://www.recoveryanswers.org/coars-2/). Through initiatives such as the virtual National Conference on Addiction Recovery Science and the monthly CoARS Showcase Series, researchers and people with lived experience are connecting to improve recovery science in real time. We invite interested readers to engage with these opportunities to contribute to this growing, collaborative field. If you would like to join the free virtual Showcase Series or learn about other opportunities, please reach out to the contact author (Ashli Sheidow).

Research in recovery support services promises exciting developments and a brighter future for individuals in recovery, their families, and communities. By continuing to integrate scientific innovation with lived expertise and translational pathways, the field can build a stronger, more equitable, and impactful recovery ecosystem for all.

## References

[1] Jones CM Noonan RK Compton WM. Prevalence and correlates of ever having a substance use problem and substance use recovery status among adults in the United States, 2018. Drug Alcohol Depend. (2020) 214:108169. doi: 10.1016/j.drugalcdep.2020.108169 32682218 PMC11060571

[2] Substance Abuse and Mental Health Services Administration. Highlights for the 2024 National Survey on Drug Use and Health (NSDUH) [Report]. U.S. Department of Health and Human Services (2025). Available online at: https://www.samhsa.gov/data/sites/default/files/NSDUH%202024%20Annual%20Release/2024-nsduh-nnr-highlights.pdf (Accessed October 21, 2025).

[3] Centers for Disease Control and Prevention. Alcohol-Related Disease Impact (ARDI) Application. National Center for Chronic Disease Prevention and Health Promotion, Division of Population Health (n.d.). Available online at: https://nccd.cdc.gov/DPH_ARDI/default/default.aspx (Accessed October 21, 2025).

[4] CDC's National Center for Health Statistics. U.S. Overdose Deaths Decrease in 2023, First Time Since 2018 [Press release]. U.S. Department of Health and Human Services (2024, May 15). Available online at: https://www.cdc.gov/nchs/pressroom/releases/20240515.html?CDC_AAref_Val= https://www.cdc.gov/nchs/pressroom/nchs_press_releases/2024/20240515.htm (Accessed October 21, 2025).

[5] Sinclair DL Chantry M De Ruysscher C Magerman J Nicaise P Vanderplasschen W. Recovery-supportive interventions for people with substance use disorders: a scoping review. Front Psychiatry. (2024) 15:1352818. doi: 10.3389/fpsyt.2024.1352818 38577404 PMC10991812

[6] Day E Pechey LC Roscoe S Kelly JF. Recovery support services as part of the continuum of care for alcohol or drug use disorders. Addiction. (2025) 120:1497–20. doi: 10.1111/add.16751 39873444 PMC12215296

[7] Hoeppner BB Simpson HV Weerts C Riggs MJ Williamson AC Finley-Abboud D . A nationwide survey study of recovery community centers supporting people in recovery from substance use disorder. J Addict Med. (2024) 18:274–81. doi: 10.1097/ADM.0000000000001285 38426533 PMC11150096

[8] Substance Abuse and Mental Health Services Administration. Table 5.1B. Substance use disorder in past year among people aged 12 or older, by age group: percentages, 2021–2024. In: *2024 National Survey on Drug Use and Health: Detailed tables. U.S. Department of Health and Human Services* (2025). Available online at: https://www.samhsa.gov/data/report/2024-nsduh-detailed-tables (Accessed October 21, 2025).

[9] Cioffi CC Hibbard PF Hagaman A Tillson M Vest N. Perspectives of researchers with lived experience in implementation science research: opportunities to close the research-to-practice gap in substance use systems of care. Implement Res Pract. (2023) 4:26334895231180635. doi: 10.1177/26334895231180635 37790184 PMC10326466

[10] Hibbard PF. From there to here: a journey through substance use disorder, prison, and recovery. J Subst Abuse Treat. (2022) 144:108922. doi: 10.1016/j.jsat.2022.108922 36327616 PMC10849090

